# Postoperative complications in the pediatric population. Malnutrition or phase angle? Which one do we use?

**DOI:** 10.3389/fnut.2024.1474616

**Published:** 2024-10-08

**Authors:** María José Díaz-Amaya, Laura Fernanda Rosales-Arreola, Jennifer Hernández-Licona, Beatriz Pérez-Guillé, Karen Ignorosa-Arellano, Silvio Carmona-Librado, José González-Zamora, Ailema González-Ortiz

**Affiliations:** ^1^Translational Research Center, Instituto Nacional de Pediatría, Mexico City, Mexico; ^2^Master Program in Clinical Nutrition, Universidad del Valle de México, Mexico City, Mexico; ^3^Department of Gastroenterology, Instituto Nacional de Pediatría, Mexico City, Mexico; ^4^Department of General Surgery, Instituto Nacional de Pediatría, Mexico City, Mexico

**Keywords:** postoperative complications, malnutrition, phase angle, pediatric surgery, body composition

## Abstract

**Background and Aims:**

Malnutrition increases post-operative risks like infections and prolonged stays. Pediatric assessment challenges require using anthropometric measurements and phase angle, which reflects body cell mass and health outcomes. Phase angle varies by maturation stages, making it crucial for pre-surgical evaluations alongside BMI. This study aimed to determine the relationship between nutritional status, phase angle, and postoperative complications in pediatric patients who underwent surgery.

**Methods:**

Prospective study with patients aged 3–17 undergoing major non-ambulatory surgery. Anthropometric measurements (weight, height, BMI *Z*-scores) hand grip strength, dietary intake and body composition via bioimpedance to assess phase angle were recorded. Postoperative complications were monitored, including surgical site infections, morbidity (pneumonia, inotropic support, infections, thromboembolism), and mortality. Surgical risks and pre- and postoperative conditions were documented.

**Results:**

After the application of the selection criteria, a total of 391 patients who underwent surgery were included; 60% (*n* = 235) were within the range of the preschool and school-age groups. During the follow-up period, 51 (13%) patients developed at least one postoperative complication, with surgical site infections being the most common. Moreover, as phase angle decreased, the length of stay (LOS) increased in all the participants. Among children aged ≤12 years old, malnutrition was a risk factor for complications [OR 3.86 (1.61–9.27 95%CI)], whereas among adolescents, phase angle served as a protective factor [OR 0.63 (0.42–0.94 95%CI)].

**Conclusion:**

Significant associations were observed between nutritional status, by BMI z-score, and post-surgical complications in younger patients. Additionally, in adolescents, the phase angle emerged as a protective factor against these complications.

## Introduction

Malnutrition is a critical concern in pediatric patients due to its significant impact on clinical outcomes. Unlike adults, where it has been identified as an independent risk factor contributing to infection development, prolonged hospital stay, post-operative complications, mortality, and high costs among surgical patients (1, 2); pediatric patients face unique challenges, including variations in growth patterns, developmental needs, and the impact of malnutrition on long-term health outcomes (3). Results indicate that up to 25% of hospitalized patients experience postoperative complications (4, 5), with the most common occurrences including superficial surgical site infections (SSIs), deep SSIs, urinary tract infections, and unplanned returns to the operating room (6). However, defining pediatric malnutrition within the context of surgical disease poses a significant challenge due to the distinct nutritional requirements of pediatric patients (7). Additionally, there is ongoing debate regarding the most effective method for assessing malnutrition in children, given that the clinical parameters established for adults often do not align well with those for pediatric malnutrition (8). Consequently, extrapolating the relationship between adult outcomes and post-surgery outcomes in the pediatric population becomes challenging.

Traditionally, malnutrition was assessed through deviations from standard growth curves, necessitating at least two data points (9). However, challenges arise when multiple data points are not readily available during the initial presentation of pediatric patients. Consequently, anthropometric growth indices such as weight-for-height, length/height, and body mass index (BMI)-for-age z scores are now recommended to evaluate nutritional status (9).

To accurately assess and categorize nutritional status in children, the Body Mass Index (BMI) must be adjusted according to age-specific *Z*-scores. The BMI *z*-score adjusts a child’s Body Mass Index for age and sex by comparing it to a reference population. A BMI *z*-score below −2.0 indicates underweight, between −2.0 and + 1.0 represents normal weight, between +1.0 and + 2.0 signifies overweight, and above +2.0 indicates obesity. These cut-off points help evaluate nutritional status in pediatrics (10).

Similarly, the incorporation of body composition assessment, which includes Phase angle (PhA), becomes crucial when evaluating these patients. Beyond the nutritional dimension, body composition appears to be indicative of clinical prognosis (11, 12). PhA, a directly measured variable in bioelectrical impedance analysis, is considered an indicator of body cell mass and the ratio of extracellular to intracellular water, and is associated with cellular integrity and function (13). PhA has shown positive correlations with various nutritional indicators, such as current weight, arm muscle area (AMA), and percentage of ideal body weight (%IBW). These correlations are especially strong in malnourished children, indicating that a lower PhA may be associated with poorer nutritional condition. In fact, it has a predictive value that exceeds that of some nutritional screening tools, such as STRONGkids (14). Assessing body composition in children and adolescents is essential for evaluating their nutritional status during growth and may impact health outcomes. Furthermore, PhA has been reported to exhibit non-linear behavior. Differences between school-aged children and adolescents are observed, potentially due to varying stages of sexual maturation. Previous studies show a correlation between BMI and PhA in the pediatric population, suggesting that both tools could be used in pre-surgical evaluation throughout life (13).

PhA is a variable derived from bioelectrical impedance analysis that has emerged as a significant indicator in the assessment of nutritional status and overall health, as it reflects body cell mass and the ratio of extracellular to intracellular water (15).

In the pediatric population, PhA increases with age, particularly after puberty, and may differ between sexes due to pubertal development. Additionally, it is positively associated with physical fitness and lean body mass (13).

Incorporating PhA into clinical assessment can provide an additional perspective beyond traditional indices, aiding in the customization of nutritional and intervention strategies to improve postoperative outcomes in the pediatric population.

Recognizing patients with nutritional deficiencies enables the development of treatment strategies aimed at achieving improved outcomes, potentially reducing the risk of postoperative mortality in future patients. Additionally, it is important to note that there is no single tool available for evaluating patients. Both PhA and BMI are generally practical and easy to apply. However, it remains unclear whether they perform equally well in school-aged children and adolescents (12). Therefore, our aim is to determine the relationship between nutritional status, PhA, and post-surgical complications in pediatric patients undergoing surgery.

## Materials and methods

### Study design

This prospective cohort study was conducted from March to November 2022, involving patients who underwent major non-ambulatory surgery. The study was carried out in a third level hospital where surgeries in different areas are performed, general surgery, orthopedic surgery, plastic surgery, urology, otorhinolaryngology, oncology, cardiology and stomatology. However, for this study only major elective surgery was considered. The inclusion criteria comprised patients aged 3–17 years, while the exclusion criteria encompassed patients scheduled for vascular access placement, closed reduction for bone fractures, ophthalmologic exploration under sedation, and non-therapeutic endoscopic procedures, as well as those who had undergone surgery within 2 months prior to evaluation. The protocol received approval from the institutional review board (IRB protocol number 2021/070). All participants provided their assent and parental or guardian-signed informed consent.

### Nutritional status

Anthropometrical measurements were recorded upon admission, before the surgery, including weight and height, obtained from participants dressed in light-weight clothing and without shoes, measured using methods previously described by the World Health Organization (WHO) (9), before the patient entered the operating room. BMI was categorized into age-*Z*-scores for patients, classifying malnutrition as: none: > −1, mild: −1 to −1.9, moderate: −2 to −2.9, severe: <−3 (9). Additionally, we categorized BMI into three categories: normal weight, overweight, and obesity, according to CDC 2022 for 2–20 years (16) and with the support with app https://peditools.org/growthpedi/.

We obtained weight and height measurements just before their surgical procedure, using the SECA scale the same scale for all patients, and the same metric ruler for height. For younger patients or those with disabilities, we used a metric ruler for stretcher and the same SECA scale used for wheelchairs. With the patients with disabilities or minors, the family members supported us.

### Body composition for phase angle

Measurements were performed by the operator using an impedance device (Bioelectric RJL Systems Quantum IV).^1^ Bioimpedance is a method used to assess body composition by measuring the resistance and reactance of body tissues to an imperceptible multifrequency electrical current. This helps determine the distribution of body fluids and tissue mass. One of the key variables obtained from bioimpedance analysis (BIA) is the PhA, which reflects the relative ratio of extracellular to intracellular water and is considered an indicator of cell membrane integrity and overall cellular health. It was measured using the previously described standardized method (15). All patients fasted for at least 4 h before the measurement and before surgery, and they were requested to remove metal objects that were in direct contact with the body and could potentially interfere with the test. Subjects were positioned in a supine posture, with arms and legs extended away from the body and palms facing downward. Electrodes were positioned in pairs on the right extremities, located at the back of the hand and foot, through which an imperceptible multifrequency current was introduced (15). After we obtaining the anthropometric and analyzer data, these were entered into the system https://www.realbia.com/interactive-online-bia/, where the “New-Pediatric” equation was selected and with that the values of fat-free mass and muscle mass were obtained.

### Outcomes

General complication was defined as the presence of the following characteristics within 30 days after surgery: death, unplanned return to the operating room, morbidity (including pneumonia, inotropic support, urinary tract infection, venous thromboembolism, respiratory failure, postoperative mechanical ventilation, and oxygen support), or SSIs (17).

Postoperative morbidity was defined as the presence of pneumonia or urinary tract infection, the requirement for inotropic support (using medications necessary to increase cardiac output), mechanical ventilation, or oxygen supplementation within the first 48 h after surgery, or the diagnosis of venous thromboembolism or renal failure (17). Regarding SSIs, characteristics of the surgical wound were assessed, including pain or tenderness, localized inflammation, redness, heat, purulent drainage, dehiscence, fever exceeding 38°C, or the presence of an abscess in the surgical wound.

### Follow-up

The primary outcome of interest was the development of postoperative complications within 30 days after surgery.

### Covariates

Upon admission, demographic characteristics and dietary intake of the enrolled children were recorded, including their age, which was grouped into pre-school children aged 3–12 years old and adolescents older than 12 years old. Surgical risk factors, including hematological disorders, neuromuscular disorders, cardiac risk factors, structural abnormalities of the central nervous system, and developmental delays, were assessed. Pre-operative conditions, including sepsis 48 h prior to surgery, mechanical ventilation, oxygen supplementation, inotropic support, and parenteral nutrition, were documented. Postoperative conditions were categorized by discharge status (outpatient or inpatient), type of surgical wound (clean, clean-contaminated, or contaminated), number of procedures, and duration of surgery in minutes.

## Statistical analysis

Values are presented as mean and standard deviation (SD) for continuous variables with a normal distribution, median (interquartile range, IQR) for non-normally distributed variables, and as a percentage of the total for categorical variables. Differences between groups were assessed using the Chi-squared test, Student’s t-test, or Mann–Whitney U test, as appropriate. We used logistic and linear regression to calculate the odds ratio of post-surgical complications associated with nutritional characteristics. Multiple imputation analyses were performed for missing data. Those variables that were potentially confounding and statistically significant in the bivariate analysis were included in the logistic regression.

A *p* value <0.05 was considered statistically significant. All statistical analyses were performed using STATA software (version 17.1; Stata Corp., College Station, TX).

## Results

During the study period, a total of 1,233 surgeries were performed, of which 404 met the criteria and were invited to participate, 12 of them accepted but their surgeries were canceled or their phone number changed, a total of 391 patients who underwent surgery in 2022 were included, with 60% (*n* = 235) being schoolchildren and preschoolers and the majority of all the sample were male (*n* = 230, 59%).

In children ≤12 years old, considering the critical stages of neurological and muscular development, neuromuscular disorders and developmental disabilities were the most common comorbidities, whereas central nervous system structural abnormalities were predominant in those >12 years old. Regarding surgical-related conditions, the study revealed that most outpatient cases (*n* = 251, 64%) were discharged. Additionally, the duration of procedures (measured in minutes) was notably extended in individuals aged >12 years, with a comparison of 70 min versus 90 min (*p*-value <0.05). This suggests that surgical procedures in adolescents may be more complex or involve longer preparation times. The most common services were general surgery, orthopedics, and plastic surgery (Table 1).

**Table 1:** General characteristics of the population.

Characteristics	All participants *n* = 391	Preschoolers and schoolers *n* = 235	Adolescents *n* = 156	*p*-value
Gender				
Female *n*, (%)	161 (41)	97 (41)	64 (41)	0.99
Surgical risk factors				
Comorbidities				
Neuromuscular disorder *n*, (%)	29 (7)	20 (9)	9 (6)	0.31
Hematological disorder *n*, (%)	21 (5)	12 (5)	9 (6)	0.78
Cardiac risk factor *n*, (%)	22 (6)	15 (6)	7 (4)	0.43
Central Nervous System structural abnormality *n*, (%)	22 (6)	10 (5)	12 (8)	0.15
Developmental delay n, (%)	25 (6.5)	19 (8)	6 (4)	0.09
Conditions associated with surgery				
Pre-surgery condition* *n*, (%)	6 (1.5)	4 (2)	2 (1)	0.99
Postoperative condition				
Postoperative stay				
In-patient *n*, (%)	140 (36)	74 (31)	66 (42)	<0.05
Out-patient *n*, (%)	251 (64)	161 (69)	90 (58)	
Type of surgical wound				
Clean *n*, (%)	336 (86)	201 (86)	135 (86)	0.88
Clean-contaminated/contaminated** *n*, (%)	140 (36)	29 (14)	21 (14)	
Number of procedures (IQR)				
One procedure	283 (72)	169 (72)	114 (73)	0.82
More than one procedure	108 (28)	66 (28)	42 (27)	
Duration of surgery in minutes, median (IQR)	80 (50–130)	70 (50–120)	90 (60–150)	<0.05
Days of preoperative stay, median (IQR)	1 (1–3)	1 (1–3)	1 (1–5)	0.47
Hospital stays (days)	0.4 (4.5)	0.2 (3.5)	0.6 (5.3)	0.36
Treating service				
General surgery *n*, (%)	97 (25)	65 (28)	32 (21)	0.14
Orthopedics *n*, (%)	100 (26)	56 (24)	44 (28)	
Plastic surgery *n*, (%)	74 (19)	38 (16)	36 (23)	
Others *** *n*, (%)	120 (30)	76 (32)	44 (28)	

^*^Sepsis 48 h prior, mechanical ventilation, oxygen supply, inotropic support, parenteral nutrition. ^**^No dirty-infected wound events. ^***^Urology, Otorhinolaryngology, Oncological surgery, Cardiology surgery, Stomatology.

By the end of the follow-up period, 51 (13%) patients had developed post-surgical complications, with surgical site infection being the most common complication (Table 2). Although the overall complication rates did not significantly differ between age groups, specific complications like surgical site infections were prevalent across all the participants.

**Table 2:** Post-operative complications.

Complications	All participants *n* = 391	Preschoolers and schoolers *n* = 235	Adolescents *n* = 156	*p*-value
Any complication *n*, (%)	51 (13)	27 (12)	24 (15)	0.26
Morbidity * *n*, (%)	10 (2.6)	5 (2)	5 (3)	0.53
Death *n*, (%)	1 (0.3)	0 (0)	1 (0.5)	0.22
Surgical site infection *n*, (%)	41 (10.5)	23 (10)	18 (12)	0.58
Unplanned return to the operating room *n*, (%)	6 (1.5)	2 (1)	4 (3)	0.18

^*^Pneumonia, inotropic support, urinary tract infection, venous thromboembolism, respiratory failure, postoperative mechanical ventilation and oxygen support were considered.

Table 3 presents the general characteristics of nutritional status and body composition according to post-surgical complications. We observe in younger participants a higher prevalence of complications in malnutrition by BMI and according to PhA it was lower in adolescents with complications (*p* < 0.05 for both). Regarding hydration status, patients with postoperative complications have a higher extracellular water, with a median of 10.9 liters compared to 8.3 liters in those without complications (*p* = 0.036).

**Table 3:** Nutritional and body composition characteristics according to complications.

Nutritional characteristics	All participants *n* = 391	Uncomplicated *n* = 340	Complications *n* = 51	*p*-value
Malnutrition by *Z*-score (yes) *n*, (%)	68 (17)	55 (16)	13 (26)	0.11
Preschoolers and schoolers *n*, (%)	45 (19)	31 (17)	10 (40)	0.013
Adolescents *n*, (%)	23 (15)	24 (15)	3 (12)	0.77
Nutritional status by BMI percentile, *n*, (%)				
Malnutrition	41 (10)	37 (11)	8 (16)	
Well nourished	226 (58)	195 (57)	31 (61)	0.08
Overweight	66 (17)	67 (20)	3 (6)	
Obesity	58 (15)	41 (12)	9 (18)	
Hand grip strength (kg), median (IQR)				
Preschoolers and schoolers				
Female	8.7 (5.8–11.4)	8.7 (6.4–11.1)	9.8 (2.4–12.4)	056
Male	7.9 (4.6–12.0)	8.1 (4.2–11.9)	7.7 (7.0–14.6)	0.72
Adolescents				
Female	19 (15.4–22.7)	18.9 (14.4–22.7)	19.8 (17.3–21.2)	0.93
Male	26.4 (19.2–33.6)	26.5 (19.8–34.2)	21.3 (13.1–30.3)	0.16
Bioelectrical impedance				
Phase angle (°), median (IQR)	5.08 (4.4–5.7)	5.1 (4.4–5.7)	5.2 (4.2–5.8)	0.67
Fat (kg), median (IQR)	12.0 (7–19.6)	11.9 (7–19.6)	15.1 (6.6–19.5)	0.38
Fat of total weight (%), mean ± SD	29.2 ± 9.9	29.3 ± 9.9	28.7 ± 10.3	0.73
FFM (kg), median (IQR)	29.7 (20.6–39.6)	28.8 (20.1–38.9)	33.4 (26.2–44.9)	0.037
SMM (kg), median (IQR)	12.4 (8.2–17.6)	11.8 (8–17.1)	14.2 (10.3–19.5)	0.087
Preschoolers and Schoolers, median (IQR)				
Female	8.4 (6.5–10.5)	8.2 (6.6–10.2)	9.2 (6.5–14.5)	0.48
Male	8.6 (6.5–11.4)	8.6 (6.5–11)	9.9 (6.7–13.1)	0.56
Adolescents, median (IQR)				
Female	15.1 (12.9–17.3)	15.1 (13.0–17.3)	13.7 (12.8–16.7)	0.97
Male	19.9 (16.0–25)	19.8 (16.0–25.2)	19.9 (17.9–22.5)	0.81
SMM of total weight (%), median (IQR)	29.0 (25.8–33.1)	28.9 (25.8–32.6)	30.4 (24.8–34.1)	0.54
SMM of FFM (%), median (IQR)	42.2 (39.1–45)	42.2 (38.8–44.9)	42.8 (40.2–45.7)	0.42
Total body water (L), median (IQR)	22.3 (16.1–28.7)	21.9 (16–27.9)	24.2 (19.4–32.4)	0.050
Intra-cellular water (L), median (IQR)	13.1 (10.8–16.7)	12.9 (10.7–16.3)	14.0 (12.4–18.9)	0.094
Extra-cellular water (L), median (IQR)	8.7 (5.6–12.2)	8.3 (5.5–12.1)	10.9 (6.7–13.6)	0.036
Dietary intake				
DEI (kcal/day), media (IQR)	1702 (1442–1899)	1,696 (1435–1913)	1723 (1549–1861)	0.50
Preschoolers and Schoolers	1,612 (1370–1831)	1,586 (1367–1825)	1,695 (1570–1856)	0.13
Adolescents	1790 (1583–1976)	1781 (1512–2004)	1783 (1549–1861)	0.44
Proteins (g/kg/day), median (IQR)	1.9 (1.27–2.84)	2.0 (1.3–2.8)	1.5 (1.2–3.1)	0.51
Preschoolers and Schoolers	2.6 (1.8–3.5)	2.7 (20–3.5)	3.0 (2.0–4.6)	0.38
Adolescents	1.3 (1.0–1.7)	1.3 (1.0–18)	1.2 (1.1–1.4)	0.28
Fat percentage, median (IQR)	33 (29–38)	33 (28.7–37.9)	33 (27–37.2)	0.58
Preschoolers and Schoolers	33.8 (29.3–38)	33.3 (29.4–38.0)	36 (31.7–40.0)	0.13
Adolescents	32.3 (27.5–37.4)	33 (28–37.5)	29 (24–35.4)	0.03

BMI, Body Mass Index; DEI, Dietary energy intake; FFM, Fat-Free Mass; SMM, Skeletal Muscle Mass.

After applying linear regression models, we identified an association between dietary fat intake and duration of surgery in younger patients (≤ 12 years old), as well as an association between length of stay (LOS) and PhA across the population (Table 4).

**Table 4:** Linear regression for association between surgery duration and hospital stay with nutritional characteristics.

	All participants		Preschoolers and schoolers		Adolescents	
	*β*. Coefficient (95% CI)	*p*-value	*β*. Coefficient (95% CI)	*p*-value	*β*. Coefficient (95% CI)	*p*-value
Duration of surgery (minutes)						
Malnutrition (yes)	7.28 (−13.55,28.44)	0.50	14.63 (−11.27,40.54)	0.27	−0.14 (−38.13,37.86)	0.99
Phase angle	1.44 (−6.19,9.06)	0.71	3.55 (−8.66,15.77)	0.57	−8.52 (−20.73,3.68)	0.37
Dietary fat/day (%)	−1.76 (−3.03, −0.49)	<0.01	−2.49 (−4.19, −7.44)	<0.01	−0.74 (−2.67, 1.19)	0.45
FFM	0.33 (−0.42, 1.07)	0.39	0.10 (−1.74, 1.94)	0.91	0.09 (−1.33, 1.51)	0.90
Hand grip strength	0.60 (−0.19–1.40)	0.14	0.95 (−0.98, 2.88)	0.33	−0.53 (−2.01, 0.95)	0.48
Hospital stay (days)						
Malnutrition (yes)	0.53 (−0.66, 1.72)	0.38	−0.49 (−1.94, 0.95)	0.50	2.37 (0.24, 4.50)	0.03
Phase angle	−0.85 (−1.26, −0.43)	<0.01	−1.18 (−0.18, −0.52)	<0.01	−1.09 (−1.77, −0.42)	<0.01
Dietary fat/day (%)	−0.05 (−0.12, 0.02)	0.19	−0.9 (−0.19, 0.01)	0.07	0.01 (−0.09, 0.12)	0.88
FFM	−0.02 (−0.06, 0.03)	0.445	−0.01 (−0.10,0.08)	0.82	−0.06 (−0.15, 0.02)	0.16
Hand grip strength	−0.01 (−0.06, 0.03)	0.59	0.01 (−0.11, 0.11)	0.98	−0.06 (−0.15, 0.02)	0.15

FFM, Fat-Free Mass. All models: Adjusted by gender, comorbidities, and dietary intake.

In the logistic regression model, for children (≤ 12 years old), malnutrition, as indicated by the z-score, was a significant risk factor for surgical site infections, morbidity, and general complications (*p* < 0.01). Conversely, for patients >12 years old, PhA emerged as a protective factor against the same post-operative complications (Table 5). All models were adjusted for gender and comorbidities, highlighting the differential significance of malnutrition and PhA in postoperative complications according to patient age (Table 5).

**Table 5:** Logistic regression for the association between perioperative complications and nutritional characteristics.

	All participants		Preschoolers and schoolers		Adolescents	
	OR (95% CI)	*p*-value	OR (95% CI)	*p-*value	OR (95% CI)	*p*-value
Surgical site infection						
Malnutrition (yes)	1.65 (0.75–3.62)	0.21	3.72 (1.42–9.69)	<0.01	0.29 (0.04–2.52)	0.27
Phase angle	0.89 (0.65–1.23)	0.49	1.24 (0.70–2.18)	0.46	0.69 (0.44–1.07)	0.10
Dietary fat/day (%)	0.98 (0.94–10.4)	0.61	1.02 (0.95–1.09)	0.57	0.94 (0.87–1.02)	0.14
FFM	1.01 (0.98–1.04)	0.48	1.01 (0.94–1.09)	0.37	1.01 (0.95–1.07)	0.82
Hand grip strength	0.99 (0.96–1.02)	0.56	1.03 (0.95–1.13)	0.53	0.95 (0.88–1.01)	0.10
Morbidity						
Malnutrition (yes)	3.63 (0.92–14.26)	0.06	14.95 (1.70–131)	0.01	1.15 (0.09–13.7)	0.91
Phase angle	0.66 (0.35–1.24)	0.19	1.05 (0.35–3.20)	0.93	0.35 (0.15–0.85)	0.02
Dietary fat/day (%)	0.96 (0.86–1.07)	0.42	1.01 (0.86–1.20)	0.83	0.91 (0.76–1.09)	0.30
FFM	1.03 (0.97–1.09)	0.30	0.83 (0.62–1.13)	0.23	1.02 (0.92–1.13)	0.72
Hand grip strength	1.03 (0.96–1.09)	0.41	0.99 (0.81–1.21)	0.94	0.98 (0.88–1.09)	0.76
General Complication						
Malnutrition (yes)	1.81 (0.89–3.67)	0.10	3.86 (1.61–9.27)	<0.01	0.51 (0.11–2.50)	0.41
Phase angle	0.84 (0.63–1.13)	0.25	1.01 (0.61–1.67)	0.97	0.63 (0.42–0.94)	0.02
Dietary fat/day (%)	0.97 (0.93–1.02)	0.22	1.01 (0.94–1.08)	0.75	0.93 (0.86–0.99)	0.04
FFM	1.02 (0.99–1.05)	0.11	1.01 (0.94–1.09)	0.71	1.01 (0.96–1.07)	0.58
Hand grip strength	1.00 (0.97–1.03)	0.99	1.00 (0.93–1.08)	0.95	0.96 (0.91–1.02)	0.23

FFM, Fat-Free Mass. All models: Adjusted by gender and comorbidities, and dietary intake.

## Discussion

This study identified that PhA is associated with hospital stay time in all the participants. We found an association between malnutrition assessed by BMI *z*-score, and postoperative complications in children under 12 years old, whit no association in older patients. Regarding, PhA and dietary fat emerged as a protective factor against postoperative complications in adolescents. These findings underscore the importance of preoperative assessment that considers both. No association founded with hand grip strength or FFM.

Moreover, tailoring interventions based on age-specific risk factors may improve the effectiveness of postoperative care. It has been observed that PhA is a valuable marker in pediatric populations, with lower PhA values associated with malnutrition in younger children. They highlighted the need for standardized PhA measurements to improve comparability and diagnostic accuracy. Their review reinforces the importance of integrating PhA into preoperative assessments (14).

It has been reported that up to 50% of patients undergoing surgery experience some complications related to the procedure (7, 18–20). An observation was made in this study that 14% of the participants experienced some form of complication within the first 30 days after surgery (all elective), with the majority originating from orthopedic and general surgery services.

Differences in the incidence of complications could be largely influenced by the characteristics of the population, the types of interventions performed, or even the types of complications reported. Disparities have been noted in the occurrence across different racial groups and age categories, with a higher prevalence observed in patients aged 12 years or younger (19).

In this study, surgical site infections exhibited the highest incidence in the overall population, showing a pattern consistent with previous findings in general surgery. However, this incidence was slightly higher than that reported for appendectomy in the pediatric population (20, 21). Additionally, a lower frequency of reoperations was observed compared to what has been reported in the pediatric population with Crohn’s disease (7).

Numerous studies have demonstrated a possible association between malnutrition and a higher risk of postoperative complications in adults (2, 7, 18). Previous study results cannot be directly extrapolated to the pediatric population, although evidence suggests that malnourished children with congenital heart disease (CHD) and those undergoing otolaryngological surgeries may experience significant deterioration in their nutritional status post-surgery (10, 22–24). While preoperative malnutrition assessed through anthropometric measures does not show a significant association with postoperative complications in elective pediatric surgeries (25), specific studies have found that parameters like mid-arm circumference z-scores and serum protein concentrations are useful in predicting certain short-term postoperative outcomes (26). Phase angle, a key indicator of body composition, has been shown to be a valuable predictor of complications in other populations (10, 14). Including phase angle in preoperative assessments could help identify patients who may benefit from appropriate perioperative nutritional interventions.

Based on the findings, our study recommends preoperative evaluations for all individuals scheduled for elective surgery. However, the use of PhA measurements may need to be tailored based on age.

Assessing nutritional status in the pediatric population presents a challenge in daily clinical practice due to the changes during this stage of life. Recently, the American Society for Parenteral and Enteral Nutrition (ASPEN) guidelines (9) recommend evaluating malnutrition in the pediatric population using the BMI-for-age *z*-score, which serves as a sensitive and useful tool to identify patients with malnutrition. Based on these guidelines, association between preoperative nutritional status and postoperative LOS has been reported (7, 27), consistent with findings from our study. Furthermore, a negative effect on intensive care unit LOS and the duration of mechanical ventilation has also been reported (24).

The BMI *z*-score alone does not fully capture the complexity of nutritional status and its impact on clinical outcomes, particularly in post-surgical processes.

PhA, on the other hand, measures cell membrane integrity and cell mass, reflecting tissue quality and overall nutritional and health status. Therefore, our study aligns with evidence showing that PhA can be a sensitive marker for malnutrition and inflammatory status, with the ability to predict complications and adverse outcomes across various populations (12, 28). PhA has been demonstrated to have a significant association with BMI and other nutritional indicators, suggesting that these two parameters could offer complementary insights into nutritional status (14).

PhA, by reflecting cellular mass integrity and body composition (29), offers a complementary perspective that may be more sensitive to changes in muscle mass and hydration status (30), which are crucial for postoperative recovery (31). In younger children, the BMI *z*-score may not fully capture fluctuations in muscle mass and hydration that are important for postoperative recovery (32). PhA, by measuring body reactance, could provide a more accurate assessment of these factors (15).

It has been suggested that an important factor such as BMI has an impact on the duration of surgery, with each additional BMI point resulting in an increase of 58.0 s in surgical time (26, 33). However, the impact of dietary factors on these outcomes has been understudied. Our study observed that dietary fat intake in children aged 12 years or under is associated with prolonged duration of surgery. This could be explained by the influence of high fat intake in the diet, which increases the BMI of patients and affects both the preparation and the surgical procedure. It also contributes to difficulty in transferring the patient to the stretcher in the operating room and in proper management of the airways. Excess fat can interfere with intraoperative surgical exposure (25).

However, there is limited evidence evaluating the effect of preoperative malnutrition and PhA in the pediatric population (27). The PhA is determined by the resistance to the current and the capacitance of the cell membrane (reactance) (15). It has been used to quantify the integrity of cell membranes and the extent of their redistribution and can be negatively influenced by various clinical conditions, such as malnutrition (14).

In adults, associations between PhA, anthropometric measurements, and muscle strength, as well as postoperative infectious complications, have been reported (34). This confirms the notion that preoperative malnutrition can serve as an independent predictor of serious complications in patients undergoing major surgery (35, 36). In this study, PhA was associated with postoperative LOS across the entire population, consistent with findings from previous studies (18, 37–42), particularly when the PhA falls below 2.7 after 2 days of hospitalization (37).

The use of PhA as an indicator of malnutrition risk should be accompanied by a thorough assessment of hydration status, specifically extracellular water expansion. The behavior of extracellular water in patients scheduled for surgery is interesting, as it could be related to chronic inflammation or pre-existing metabolic stress (43, 44). A study in patients with cancer and sarcopenia suggests that increased ECW indicates an inflammatory state, which is associated with poorer clinical outcomes (45).

According to Lukaski et al. (46), PhA is a marker of cell membrane integrity and cellular health, but its reliability as a predictor of malnutrition is compromised by fluid shifts resulting from inflammation and disease-related malnutrition. Similarly, Bellido et al. (47) emphasize that standardizing PhA measurements and incorporating hydration assessments are crucial for accurate risk evaluation and monitoring of nutritional status. This consideration is particularly relevant in postoperative settings, where inflammation and fluid imbalance can significantly impact recovery and the risk of complications. Therefore, integrating hydration status into BIA evaluations can enhance the accuracy of predicting malnutrition and guide more effective patient management strategies.

For the analysis of nutritional status, it is important to use the PhA, which reflects changes in the cell membrane and alterations in the fluid balance. It has been observed that measuring the preoperative PhA helps in predicting the occurrence of postoperative infections (48), with a decrease noted in instances of disease, inflammation, or malnutrition (49) caused by microorganisms after major surgery (48). Malnutrition is a very important factor in immune dysfunction, affecting the body’s ability to fight infections (48). It also influences wound healing; for instance, obesity can increase the risk of rupture or infectious complications, while a diet with low protein and energy intake can delay proper healing (33, 50). Because the PhA serves as a marker for both the quantity and quality of soft tissue mass, many authors consider it a useful marker of nutritional status and the strongest predictor of complications (14).

As part of the strengths of this study, the prospective design allows real-time data collection, which enhances data accuracy and minimizes retrospective biases. The inclusion of pediatric patients aged 3–17 provides a comprehensive insight into the association between nutritional status and postoperative complications across various developmental stages. Assessing nutritional status through parameters such as BMI, *z*-score, muscle strength, and PhA offers a thorough evaluation of patients’ physical condition. Considering clinical risk factors, such as neuromuscular disorders and central nervous system structural abnormalities, strengthens the validity of the results by accounting for significant variables that could influence postoperative complications. The limitations of the study include its sample size, which may potentially restrict the generalization of the findings to a broader population. Future research with larger cohorts could validate and strengthen the obtained results. Additionally, the 30-day follow-up post-surgery may not capture long-term complications. It is important to take these data with caution, it is necessary to do future studies with a specific analysis for those patients with neurological or muscular impairment.

All the findings in this study provide a comprehensive overview of the surgical landscape, patient demographics, and postoperative outcomes. Tailoring interventions based on age-specific risk factors and understanding the association between nutritional status and surgical outcomes are crucial for improving patient care and optimizing healthcare resources. Malnutrition is an important predictor of serious complications in patients undergoing major surgery. The PhA serves as a valuable tool for assessing the nutritional status of surgical patients and for predicting the risk of postoperative complications.

## Conclusion

Malnutrition, as indicated by *z*-scores, is an important predictor of postoperative complications in preschoolers and school-age children who underwent surgery, whereas PhA proves useful in predicting complications in adolescents. PhA was a predictor of length of hospital stay in all the participants. Personalized nutritional and body composition assessments tailored to age are essential for improving outcomes for patients undergoing surgery. This research highlights the need for personalized assessment of nutrition and body composition based on age in pediatric patients undergoing surgery, which could significantly improve outcomes and patient recovery.

## Data Availability

The raw data supporting the conclusions of this article will be made available by the authors, without undue reservation.

## Footnotes

^1^ https://cdn.shopify.com/s/files/1/0829/4989/8550/files/quantum_4-manual1.pdf?v=1694476372
